# The Effects of the Use of Patient-Accessible Electronic Health Record Portals on Cancer Survivors’ Health Outcomes: Cross-sectional Survey Study

**DOI:** 10.2196/39614

**Published:** 2022-10-24

**Authors:** Piper Liping Liu, Xinshu Zhao, Jizhou Francis Ye

**Affiliations:** 1 Department of Communication Faculty of Social Sciences University of Macau Taipa Macao

**Keywords:** electronic health record, patient-centered care, health self-efficacy, cancer survivors, physical health, psychological health

## Abstract

**Background:**

In the past decade, patient-accessible electronic health record (PAEHR) systems have emerged as an important tool for health management both at the hospital level and individual level. However, little is known about the effects of PAEHR portals on the survivorship of patients with chronic health conditions (eg, cancer).

**Objective:**

This study aims to investigate the effects of the use of PAEHR portals on cancer survivors’ health outcomes and to examine the mediation pathways through patient-centered communication (PCC) and health self-efficacy.

**Methods:**

Data for this study were derived from the Health Information National Trends Survey (HINTS 5, Cycle 4) collected from February 2020 to June 2020. This study only involved respondents who reported having been diagnosed with cancer (N=626). Descriptive analyses were performed, and the mediation models were tested using Model 6 from the SPSS macro PROCESS. Statistically significant relationships among PAEHR portal use, PCC, health self-efficacy, and physical and psychological health were examined using bootstrapping procedures. In this study, we referred to the regression coefficients generated by min-max normalization as percentage coefficients (*b_p_*). The 95% bootstrapped CIs were used with 10,000 resamplings.

**Results:**

No positive direct associations between PAEHR portal use and cancer survivors’ health outcomes were found. The results supported the indirect relationship between PAEHR portal use and cancer survivors’ psychological health via (1) PCC (*b_p_*=0.029; *β*=.023, 95% CI .009-.054), and (2) PCC and health self-efficacy in sequence (*b_p_*=0.006; *β*=.005, 95% CI .002-.014). Besides, the indirect association between PAEHR portal use and cancer survivors’ physical health (*b_p_*=0.006; *β*=.004, 95% CI .002-.018) via sequential mediators of PCC and health self-efficacy was also statistically acknowledged.

**Conclusions:**

This study offers empirical evidence about the significant role of PAEHR portals in delivering PCC, improving health self-efficacy, and ultimately contributing to cancer survivors’ physical and psychological health.

## Introduction

Cancer is among the leading causes of death worldwide, accounting for about 10 million deaths in 2020 [1]. In 2021, 1.9 million new cancer cases were diagnosed and over 600,000 cancer deaths were estimated in the United States [2]. Due to the growing and aging population as well as increases in early diagnoses and advances in cancer treatments, the number of cancer survivors continues to increase [3]. According to the National Cancer Institute, “An individual is considered a cancer survivor from the time of diagnosis, through the balance of his or her life” [4]. Cancer is viewed as a chronic illness, and cancer survivors face ongoing health challenges that call for unique and long-term survivorship care. This is because physical problems such as functional disability and impairment and psychological disorders due to illness and aggressive treatments might persist throughout cancer survivors’ lifetime [3,5]. As such, delivering high-quality and long-term health care for cancer survivors becomes a major challenge facing public health.

The maintenance of long-term cancer treatment plans requires effective patient-provider communication and coordination of cancer survivorship care [6,7]. Health care information technology has brought about a massive change in cancer care. The transition to patient-accessible electronic health record (PAEHR) systems has changed the way patients and providers engage in health care by facilitating access to patient information (eg, test results) [8], allowing timely and efficient patient-provider communication [9], reducing medical errors [10], educating patients with accessible and affordable health materials [11], and enhancing the privacy and security of patient data [12]. Therefore, researchers generally agree that PAEHR portals have the potential to improve health through evidence-based medicine and effective care coordination [13]. For instance, Wani and Malhotra [14] provided empirical evidence supporting that the assimilation of PAEHRs at a hospital-wide level can help deliver quality care and services, which in turn improve patients’ health outcomes. A systematic review conducted by Kruse et al [13] identified a variety of facilitators of PAEHRs that can improve population health, including the enhancement in productivity/efficiency, the increase in the quality of patient data, and more flexible data management. Nevertheless, the majority of existing studies have inevitably investigated the PAEHR system from perspectives on professionals’ innovation adoption [15] or organizational management [16]. There remains a paucity in the literature on the use of PAEHR portals and health outcomes from patient perspectives. To address this literature gap, our study aims to investigate how PAEHR portal use influences cancer survivors’ health outcomes.

The Chronic Care Model (CCM) provides a framework for understanding the mechanisms through which health care provided via PAEHR portals influences patients’ health outcomes [17]. Six key interdependent components of CCM that are essential for care delivery have been identified: (1) health system support, (2) delivery system design, (3) clinical information systems, (4) community resources, (5) decision support, and (6) self-management support. Researchers suggest that the PAEHR portal may be a prominent tool that incorporates the key elements of CCM and determines the success of care delivery and health management [18]. CCM relies on the use of health information technology for both public and private health care systems to facilitate the provision of longitudinal and patient-centered care, improve patient engagement, and empower patients with self-care skills to manage chronic illness [18,19]. Gee et al [19] proposed a revised CCM—eHealth enhanced CCM (eCCM)—and explicated that the use of eHealth technologies can help improve chronic care (eg, through patient-centered communication [PCC], clinical decision support, information provision, health education). Consequently, experienced PAEHR users have higher health self-efficacy and can achieve improved health outcomes [19].

Proponents of the eCCM contend that eHealth adoption, referred to in this study as PAEHR portal use, is likely to impact health outcomes through indirect pathways, which comprise proximal outcomes (eg, effective patient-provider communication) of eHealth that then influence health or that contribute to intermediate outcomes (eg, health self-efficacy) that lead to improved distal health outcomes [19]. Rathert et al [20] provide tentative support for the serial mediation effect of PCC and health self-efficacy in the relationship between PAEHR portal use and health outcomes. PCC is about delivering health care that relies upon effective communication and empathy to meet individual patient preferences, needs, and values [21,22]. Health self-efficacy refers to people’s beliefs regarding one’s capabilities to execute the courses of action to improve health [23]. There is a general consensus that the PAEHR is more than a tool that serves for patient data collection and information exchange. It is a “third agent” during patient care encounters that essentially improves PCC [20,24]. For example, patients who used PAEHR portals prior to doctor visits reported that communication with their physicians improved considerably [25]. This is because the patient data in the PAEHR system enables providers to monitor patients’ symptoms and medication adherence [26]. Physicians thus would spend much time and pay more attention to patients during clinical encounters [27]. Meanwhile, patients who used PAEHR portals perceived more PCC, as they felt empowered to ask questions or offer comments regarding their health problems [24,28]. By this token, PAEHR portal use and PCC can facilitate patients’ management of their health and should eventually contribute to health improvement [20,21,29]. Street et al [29] proposed a pathway model of health communication and suggested that, in most cases, PCC affects patient health through a more indirect route via an intermediate outcome of communication, such as health self-efficacy. It is understandable that PCC can increase patients’ health self-efficacy because providers’ clear explanations and expressions of support could increase patient knowledge and shared understanding, motivate patients to follow through with treatment recommendations, and thus improve patients’ confidence in self-care management.

**Figure 1:**
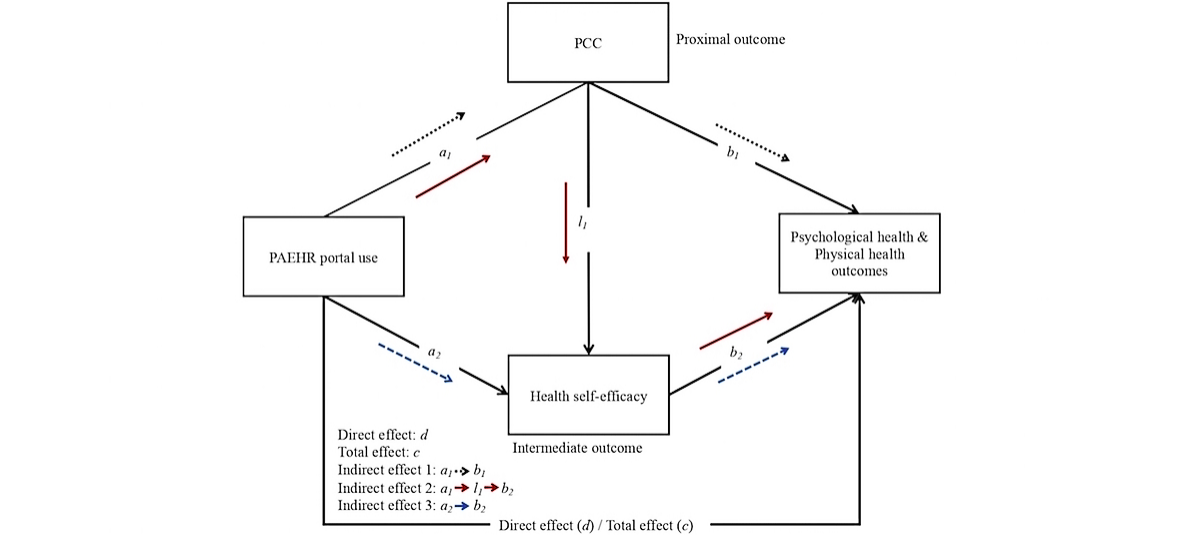
Pathways between patient-accessible electronic health record portal use and health outcomes. *a*_1_, *a*_2_, *b*_1_, *b*_2_, and *l*_1_ indicate the pathways and the effects. PAEHR: patient-accessible electronic health record; PCC: patient-centered communication.

Following this line, 2 mediators—PCC and health self-efficacy—were conceptualized as the proximal and intermediate outcomes of PAEHR portal use, respectively. Previous research that examined related variables has provided empirical support. For instance, Madhavan et al [30] found that due to the transportability and interoperability, effective use of PAEHR contributes to improved PCC, which plays a cardinal role in cancer survivors’ health management. Guo et al [31] found that eHealth adoption (eg, seeking web-based health information and using health apps) was significantly associated with improved self-care skills, which further led to more positive self-rated health among Taiwanese patients with chronic diseases [31]. Liu and Yeo [22] conceptualized a framework, suggesting that web-based patient-provider communication via eHealth technologies may improve patients’ quality of life through sequential mediators of patient-centered care and health management skills. Building on prior research, this study aims to examine the relationships among cancer survivors’ PAEHR portal use, PCC, health self-efficacy, and health outcomes. Moreover, the mediation roles of PCC and health self-efficacy were tested. Thus, the following direct and indirect relationships between PAEHR portal use and cancer survivors’ health outcomes (see Figure 1) were proposed:

Hypothesis 1: PAEHR portal use is positively related to cancer survivors’ health outcomes.

Hypothesis 2: PCC mediates the relationship between PAEHR portal use and cancer survivors’ health outcomes.

Hypothesis 3: Health self-efficacy mediates the relationship between PAEHR portal use and cancer survivors’ health outcomes.

Hypothesis 4: PCC and health self-efficacy sequentially mediate the relationship between PAEHR portal use and cancer survivors’ health outcomes.

## Methods

### Study Design and Sample Population

Data for this study were derived from the Health Information National Trends Survey (HINTS 5, Cycle 4) collected from February 2020 to June 2020. HINTS is administered by the National Cancer Institute in the United States to collect nationally representative data about American adults’ access to health-related information, health behaviors, and health outcomes. The survey design and sampling procedures for HINTS have been explicated extensively in previous research [32]. The final sample of HINTS 5, Cycle 4 consisted of 3865 respondents (response rate=36.7%) of the 10,531 participants. This study only involved respondents who reported having been diagnosed with cancer (N=626).

### Ethical Considerations

This study used secondary data. The HINTS data meet strict ethical standards and have obtained ethics approval. Informed consent has been obtained from all participants, and all methods were carried out in accordance with relevant guidelines and regulations.

### Measures

PAEHR portal use was measured by asking respondents whether they had accessed patient portals of PAEHR in the past year for certain eHealth activities [33]. Three items were included: “Look up test results,” “securely message health care provider and staff,” and “download health information to computer or mobile device.” Responses were dichotomous (no=0, yes=1) and added up to represent PAEHR portal use (mean 1.726, SD 0.575).

PCC consisted of 7 statements that assessed patients’ perceptions of communication with all doctors, nurses, or other health professionals in the past 12 months [21,34]. A 4-point Likert scale (1=always, 4=never) was used. Responses to the 7 statements were reversely coded and averaged to create the index of PCC, and higher values represent high levels of PCC (mean 3.414, SD 0.607; Cronbach α=.93).

Health self-efficacy was measured using 1 item to assess one’s ability to take care of his/her health on a 5-point scale from 1 (completely confident) to 5 (not confident at all) [23]. Respondents’ answers were reversely scored, and a higher score represented a higher level of health self-efficacy (mean 3.804, SD 0.812).

Physical health was measured by 4 items on comorbidities, drawn from prior research of similar measures [35]. Respondents were asked whether they had been told by a doctor or another health professional that they had medical conditions such as (1) diabetes or high blood sugar; (2) high blood pressure or hypertension; (3) a heart condition such as heart attack, angina, or congestive heart failure; and (4) chronic lung disease, asthma, emphysema, or chronic bronchitis. Responses to these items were dichotomous (no=0, yes=1). The answers were added up, and a higher value indicated better physical health (mean 2.748, SD 1.082).

Psychological health was measured by 4 items derived from previous research [36]. Sample items included “feeling down, depressed, or hopeless” and “feeling nervous, anxious, or on edge.” The 4 items were measured on a 4-point scale (1=nearly every day to 4=not at all) and averaged to form a composite score representing psychological health (mean 3.502, SD 0.706; Cronbach α=.88). A higher value suggests better psychological health. The descriptive details of the focal variables are shown in Tables 1-4.

The control variables included demographics such as age, gender (male=1, female=0), education (less than 8 years=1, postgraduate=7), annual household income (US $0-9999=1, US $200,000 or more=9), and race (non-Hispanic White=1, others=0).

**Table 1:** Descriptive statistics of the patient-accessible electronic health record portal use and physical health of the participants (N=626).

		Yes	No	Nonvalid
**Patient-accessible electronic health record portal use, n (%)**				
	Look up test results	252 (40.3)	36 (5.8)	338 (53.9)
	Securely message health care provider and staff	176 (28.1)	110 (17.6)	340 (54.3)
	Download health information to computer or mobile device	68 (10.9)	218 (34.8)	340 (54.3)
**Physical health, n (%)**				
	Diabetes or high blood sugar	176 (28.1)	440 (70.3)	10 (1.6)
	High blood pressure or hypertension	374 (59.7)	244 (39)	8 (1.3)
	A heart condition such as heart attack, angina, or congestive heart failure	91 (14.5)	527 (84.2)	8 (1.3)
	Chronic lung disease, asthma, emphysema, or chronic bronchitis	132 (21.1)	486 (77.6)	8 (1.3)

**Table 2:** Descriptive statistics of patient-centered communication (N=626).

Patient-centered communication	Always, n (%)	Usually, n (%)	Sometimes, n (%)	Never, n (%)	Nonvalid, n (%)
Give you the chance to ask all the health-related questions you had	393 (62.8)	142 (22.7)	39 (6.2)	3 (0.5)	49 (7.8)
Give the attention you needed to your feelings and emotions	279 (44.6)	185 (29.6)	83 (13.3)	23 (3.7)	56 (8.9)
Involve you in decisions about your health care as much as you wanted	324 (51.8)	180 (28.8)	65 (10.4)	7 (1.1)	50 (7.9)
Make sure you understood the things you needed to do to take care of your health	362 (57.8)	169 (27)	43 (6.9)	3 (0.5)	49 (7.8)
Explain things in a way you could understand	366 (58.5)	164 (26.2)	43 (6.9)	3 (0.5)	50 (7.9)
Spend enough time with you	292 (46.6)	193 (30.8)	73 (11.7)	17 (2.7)	51 (8.2)
Help you deal with feelings of uncertainty about your health or health care	260 (41.5)	191 (30.5)	90 (14.4)	29 (4.6)	56 (9)

**Table 3:** Descriptive statistics of health self-efficacy (N=626).

Health self-efficacy	Completely confident	Very confident	Somewhat confident	A little confident	Not confident at all	Nonvalid
How confident are you about your ability to take good care of your health, n (%)	111 (17.7)	318 (50.8)	159 (25.4)	28 (4.5)	6 (1)	4 (0.6)

**Table 4:** Descriptive statistics of psychological health (N=626).

Psychological health	Nearly every day, n (%)	More than half the day, n (%)	Several days, n (%)	Not at all, n (%)	Nonvalid, n (%)
Little interest or pleasure in doing things	31 (5)	53 (8.5)	123 (19.6)	404 (64.5)	15 (2.4)
Feeling down, depressed, or hopeless	18 (2.9)	31 (5)	122 (19.5)	436 (69.6)	19 (3)
Feeling nervous, anxious, or on edge	30 (4.8)	29 (4.6)	163 (26)	389 (62.1)	15 (2.5)
Not being able to stop or control worrying	29 (4.6)	44 (7)	112 (17.9)	424 (67.7)	17 (2.8)

### Data Analysis

Data analysis was performed using SPSS version 26 (IBM Corp). First, the MEAN () function was used to compute the mean of multiple-item variables that at least one item has a valid value or single-item variables that have valid values. Otherwise, the cases were considered missing in the following analysis. Besides, as a complementary technique, min-max normalization [37] was introduced to compare the estimates of all the paths in the mediation model. Specifically, all research variables were converted into a common measurement scale of 0 to 1. For example, we can subtract 1 from a 5-point rating to adjust the scale to start at 0 and then divide it by 4 to compress the scale. In this study, we referred to the regression coefficients generated by min-max normalization as percentage coefficients (*b_p_*) [38,39]. Second, the mean substitution was used for all missing cases. Third, descriptive statistics was analyzed. Fourth, the mediation models were tested using Model 6 from the SPSS macro PROCESS; statistically significant relationships among PAEHR portal use, PCC, health self-efficacy, and physical and psychological health were examined using bootstrapping procedures. The 95% bootstrapped CIs were used with 10,000 resamplings.

## Results

The mean age of the cancer survivors was 67.46 (SD 13.19; range 19-104) years. There were more female respondents (370/626, 59.1%) than male respondents (256/626, 40.9%). The majority of the participants had received some college education (405/626, 64.7%), were non-Hispanic White (428/626, 68.4%), and had annual household income between US $35,000 and US $74,999 (259/626, 41.4%). The detailed demographic information is summarized in Table 5.

**Table 5:** Sample population characteristics (N=626).

Characteristic			Value
Age in years, mean (SD)			67.46 (13.19)
**Gender, n (%)**			
	Male	256 (40.9)	
	Female	370 (59.1)	
**Education, n (%)**			
	Less than 8 years of education	14 (2.2)	
	8-11 years of education	29 (4.6)	
	12 years of education or completed high school	132 (21.1)	
	Post high school training other than college	46 (7.3)	
	Some college	143 (22.8)	
	College graduate	145 (23.2)	
	Postgraduate	117 (18.7)	
**Annual income (USD), n (%)**			
	0-9999	33 (5.3)	
	10,000-14,999	34 (5.4)	
	15,000-19,999	37 (5.9)	
	20,000-34,999	79 (12.6)	
	35,000-49,999	87 (13.9)	
	50,000-74,999	172 (27.5)	
	75,000-99,999	58 (9.3)	
	100,000-199,999	94 (15)	
	200,000 or more	32 (5.1)	
**Race, n (%)**			
	Non-Hispanic White	428 (68.4)	
	Others	198 (31.6)	

Hypothesis 1 posited that PAEHR portal use is positively related to cancer survivors’ health outcomes. Table 6 shows that there was no significant direct association between PAEHR portal use and cancer survivors’ health outcomes, irrespective of the physical or psychological health. Thus, hypothesis 1 was not supported.

Hypothesis 2 predicted that PCC mediates the relationship between PAEHR portal use and cancer survivors’ health outcomes. As depicted in Table 6, PAEHR portal use was significantly and positively associated with PCC (*b*_p_=0.131; *β*=.125, 95% CI .048-.214; *P*=.002) in the 2 models. Meanwhile, PCC was positively associated with cancer survivors’ psychological health (*b*_p_=0.270; *β*=.269, 95% CI .258-.461; *P*<.001). No significant relationship between PCC and cancer survivors’ physical health was acknowledged. The results indicated that PCC indeed mediated the relation between PAEHR portal use and cancer survivors’ psychological health (*b*_p_=0.029; *β*=.023, 95% CI .009-.054), whereas the counterpart effect failed to pass the statistical threshold (95% CI contained zero) for physical health. Hypothesis 2 was partially supported.

Hypothesis 3 predicted that PAEHR portal use might increase cancer survivors’ health outcomes through the mediation of association with health self-efficacy. The mediation effects in the 2 models were statistically unacknowledged. Thus, hypothesis 3 was not supported.

Hypothesis 4 predicted that PAEHR portal use will be related to cancer survivors’ health outcomes through the serial mediation of PCC and health self-efficacy. As shown in Table 6, the indirect relationship between PAEHR portal use and cancer survivors’ physical health (*b*_p_=0.006; *β*=.004, 95% CI .002-.018) and between PAEHR portal use and psychological health (*b*_p_=0.006; *β*=.005, 95% CI .002-.014) via sequential mediators of PCC and health self-efficacy were statistically acknowledged, thereby supporting hypothesis 4.

**Table 6:** Mediation models^a^.

			*b_p_^b^*		*β*		SE	95% CI		*P* value^c^
**Dependent variable: Psychological health (Model 1)**										
	PAEHR^d^→PCC^e^ (*a*_1_ path)	0.131		.125		.042		.048 to .214	.002	
	PAEHR→Health self-efficacy (*a*_2_ path)	0.022		.021		.055		–.078 to .137	.59	
	PCC→Health self-efficacy (*l*_1_ path)	0.270		.269		.052		.258 to .461	<.001	
	PCC→Psychological health (*b*_1_ path)	0.217		.186		.046		.127 to .306	<.001	
	Health self-efficacy→Psychological health (*b*_2_ path)	0.181		.156		.034		.068 to .202	<.001	
	PAEHR→Psychological health (direct effect, *d* path)	–0.016		–.013		.046		–.108 to .075	.73	
	PAEHR→Psychological health (total effect, *c* path)	0.023		.018		.048		–.072 to .117	.64	
	PAEHR→PCC→ Psychological health (indirect effect, *a*_1_x*b*_1_)	0.029		.023		.012		.009 to .054	N/A^f^	
	PAEHR→PCC→ Health self-efficacy→Psychological health (indirect effect, *a*_1_x*b*_2_x*l*_1_)	0.006		.005		.003		.002 to .014	N/A	
	PAEHR→Health self-efficacy→Psychological health (indirect effect, *a*_2_x*b*_2_)	0.004		<.001		.008		–.012 to .020	N/A	
**Dependent variable: Physical health (Model 2)**										
	PAEHR→PCC (*a*_1_ path)	0.131		.125		.042		.048 to .214	.002	
	PAEHR→Health self-efficacy (*a*_2_ path)	0.022		.021		.055		–.078 to .137	.59	
	PCC→Health self-efficacy (*l*_1_ path)	0.270		.269		.052		.258 to .461	<.001	
	PCC→Physical health (*b*_1_ path)	0.013		.010		.070		–.120 to .154	.81	
	Health self-efficacy→Physical health (*b*_2_ path)	0.168		.126		.052		.066 to .270	.001	
	PAEHR→Physical health (direct effect, *d* path)	–0.032		–.023		.071		–.183 to .096	.55	
	PAEHR→Physical health (total effect, *c* path)	–0.021		–.015		.071		–.168 to .112	.69	
	PAEHR→PCC→Physical health (indirect effect, *a*_1_*→b*_1_)	0.002		.001		.011		–.020 to .024	N/A	
	PAEHR→PCC→Health self-efficacy→Physical health (indirect effect, *a*_1_*→b*_2_*→l*_1_)	0.006		.004		.004		.002 to .018	NA	
	PAEHR→Health self-efficacy→Physical health (indirect effect, *a*_2_*→b*_2_)	0.004		.003		.010		–.015 to .026	N/A	

^a^a_1_, a_2_, b_1_, b_2_, and l_1_ in this table indicate the pathways between patient-accessible electronic health record portal use and health outcomes and the effects.

^b^Regression coefficient generated by min-max normalization as percentage coefficient.

^c^*P* values are not computed for bootstrapped indirect effects.

^d^PAEHR: patient-accessible electronic health record.

^e^PCC: patient-centered communication.

^f^N/A: not applicable.

## Discussion

### Principal Findings

In light of the existing literature on the robust salutary effects of PAEHR portals on patient health, our study examined the effects of PAEHR portal use on cancer survivors’ health outcomes as well as the mediating roles of PCC and health self-efficacy. The results of our study indicated that the significant effect of PAEHR portal use on cancer survivors’ physical and psychological health was indirect through the mediated associations with PCC and health self-efficacy.

The direct association between PAEHR portal use and cancer survivors’ health outcomes is not acknowledged in this study. The findings of our study emphasize the mediation mechanisms through which the PAEHR portal use exerts an influence on cancer survivors’ physical and psychological health, which were in accordance with that reported in previous research that theorizes the process through which PAEHR may impact patient health [20]. Rathert et al’s [20] and Street et al’s [29] pathway models provide the needed theoretical foundation for this study, supporting that several steps must occur for health improvement to be influenced by cancer survivors’ PAEHR portal use. First, PAEHR portals serve as a tool that facilitates patient-provider communication. Physicians should incorporate PAEHR systems to provide PCC that supports patients in making informed health care decisions that are consistent with their needs, values, and preferences. Unless PCC is improved, PAEHR portal use will not increase patients’ health self-efficacy and improve their health outcomes. Although previous research has identified the association between PAEHR and patient health, we investigated the mediating mechanisms (the process) through which PAEHR impacts patient health.

PCC and health self-efficacy were identified as the intrinsic and extrinsic factors of PAEHR, respectively, that help explain how PAEHR portal use influences patients’ health outcomes. The results of our study suggest that PCC can partially mediate the relationship between PAEHR portal use and cancer survivors’ psychological health. The mediation results indicated that the more cancer survivors use the PAEHR portals to stay informed about their health and communicate with health care professionals, the more likely they are to perceive PCC, which in turn results in more positive psychological health. A plausible reason is that the increasing accessibility to health professionals and patient information facilitated by PAEHR systems may enhance patient involvement in their health care decision-making [40]. Through PAEHR portals, cancer survivors are likely to be informed about their health status, be well educated with adequate health information, and have convenient access to health care professionals for medical guidance [41]. As a result, patients feel more engaged in PCC, which helps better understand their health and motivate them to stay positive and improve their psychological health [42-44]. However, PCC has no mediation effect between PAEHR portal use and cancer survivors’ physical health. This might be because the research sample of this study consisted of 626 cancer survivors with an average age >60 years, and they were likely to have inferior health status. PCC could not improve physical health unless patients were equipped with the necessary health skills. This assumption was supported by the sequential mediation effect of PCC and health self-efficacy between PAEHR portal use and cancer survivors’ health outcomes.

The results of our study showed that PCC is positively associated with health self-efficacy, and higher levels of health self-efficacy can enhance cancer survivors’ physical and psychological health. This finding was consistent with prior research, suggesting that PCC may empower patients, help increase their self-care skills, and provide the needed information and support to facilitate patients’ health management [45,46]. Furthermore, improved health self-efficacy can help people take care of their physical and psychological health, and this finding was congruent with previous findings [47-49]. Our results provide empirical evidence of the indirect effect of PAEHR portal use on cancer survivors’ health outcomes through PCC and health self-efficacy.

### Comparison With Prior Work

Our study in comparison with previous work has heuristic value for public health research in several ways. First, the findings of our study offer empirical support for eCCM [19] and Rathert et al’s [20] pathway model in understanding the process through which PAEHR impacts patient health. Second, this study extends the current literature by investigating the usability of eHealth technologies in delivering longitudinal survivorship care for patients with chronic diseases as well as examining the mediation roles of PCC and health self-efficacy. Our findings stressed PCC as the salient intrinsic factor of PAEHR that helps improve patients’ health self-efficacy and prompts them into action to maintain their health. The mediation effects provide a more nuanced understanding of the mechanisms underlying the association between PAEHR portal use and patients’ health outcomes. This model was established in several hypotheses by which the assumptions have been shown tenable. This study thus helps consolidate past research on the relationships between PAEHR portal use and patients’ physical and psychological health.

This study also has important practical implications. First, given the important role of electronic means for health management, multifaceted strategies should be implemented to promote the assimilation of PAEHR at both institutional and individual levels. For example, through patient education and support, patients can gain knowledge about PAEHR and be encouraged to integrate PAEHR into their health care in everyday life. Besides, we should also encourage medical professionals to engage in PAEHR systems to provide customized health care services. For example, a medical professional can provide detailed explanations for certain clinical decisions through PAEHR portals, and patients can access and revisit the messages that can facilitate their self-care practices [50]. Second, considering the significant role of PAEHR portals, we should continue to develop information technology infrastructure to improve the accessibility of high-quality and long-term survivorship care. For example, patients who live remotely with low-speed internet and people who have poor internet skills may not benefit from the convenience and great efficiency brought by the internet for medical consultations [47]. Thus, information and communication technology companies should expand high-speed internet provisions to the other regions and deliver benefits to more people and communities. In addition, we should provide continuous support to help individuals overcome the barriers encountered in using PAEHR portals for health management [51]. Third, strict policies for web-based health service regulation should be implemented to protect patients’ information and to ensure a safe PAEHR environment. In parallel with the governmental measures, it is equally important to educate patients about their rights to access health data and responsibilities for personal information security. Fourth, considering the effect of PCC, it is important to help patients more actively participate in health consultations as well as provide training to physicians in delivering empathetic, mindful, informative, and patient-centered care.

### Limitations and Directions for Future Research

Several limitations of this study should be noted. First, owing to the cross-sectional design of HINTS, we know little about the causal inferences of relationships examined in this study. Further research should collect panel data or use experimental research designs to better understand the relationships among PAEHR portal use, PCC, health self-efficacy, and health outcomes. Second, according to CCM and eCCM, there are 6 key components of eHealth technologies for care delivery, such as health system support and delivery system design. However, PAEHR portal use in this study was measured using 3 items, that is, patients’ past experience in PAEHR portal use for checking test results, patient-provider communication, and health information acquisition. We know little about the influence of other aspects of PAEHR portal use. To our knowledge, no study has examined the usability of PAEHR system design and how it impacts patient-provider communication and patients’ health maintenance. Besides, PAEHR portal use was examined as an integrated concept, and we hardly know how different types of PAEHR portal usage may affect patient health differently. Based on this study, future research should take into account the different use dimensions of PAEHR systems or the different types of PAEHR portal usage and compare their different influences. Third, PCC and health self-efficacy were identified as the mediators in the relationship between PAEHR portal use and cancer survivors’ health outcomes. Other potential interveners might be overlooked. Researchers should further extend the model and identify other mediators (eg, knowledge) or moderators (eg, health literacy, digital literacy) that significantly influence PAEHR portal users’ health-related outcomes. Fourth, the research findings of our study might be impacted by sampling bias. For example, more than half of the respondents were aged between 60 years and 80 years (mean 67.46 years) and had at least completed some college education. It is recommended that a more representative sample be analyzed to better understand the full range of cancer survivors’ PAEHR portal use. Moreover, our study focused on cancer survivors, and the results may not be generalizable to other populations. PAEHR portals can likely be helpful and useful for people with other chronic conditions such as diabetes and asthma. Thus, researchers should replicate this work in other populations to obtain more tentative evidence, thereby supporting the positive association between PAEHR portal use and health outcomes.

### Conclusion

This study offers empirical evidence on the influence of PAEHR portal use on cancer survivors’ physical and psychological health. Although electronic technologies have been widely applied in health care settings, the adoption rate of PAEHR among patients remains low. This study suggests that PAEHR portal use is vital in delivering longitudinal survivorship care for cancer survivors. In particular, the influence of PAEHR portal use on health outcomes may be indirect through the mediated associations with PCC care and health self-efficacy. Understanding these relationships can help increase the use of PAEHR portals, promote PCC, enhance patients’ health self-efficacy, and eventually improve their physical and psychological health.

## Abbreviations

CCM: Chronic Care Model
eCCM: eHealth enhanced Chronic Care Model
HINTS: Health Information National Trends Survey
PAEHR: patient-accessible electronic health record
PCC: patient-centered communication

## Data Availability

The data sets generated and analyzed during this study are publicly available on [52]. All data and materials comply with field standards.
